# Rapid increase in breast volume in a 14-year old

**DOI:** 10.11604/pamj.2013.16.85.3489

**Published:** 2013-11-08

**Authors:** Abakka Sanae, Ansari Chenguiti Anas

**Affiliations:** 1 Department of Obstetrics, Gynaecology and High risk Pregnancies, Maternity Hospital Souissi – Ibn Sina teaching hospital of Rabat-Salé, Morocco

**Keywords:** Juvenile gigantomastia, breast, macromastia

## Image in medicine

This 14 year-old girl presented with a 2-month history of massive increase in breast volume. She was peripubertal and became homebound ever since. On examination, she had an important bilateral and symmetric breast ptosis with widening of the nipple-areolar complex. No breast nodule, axillary lymphadenopathy or galactorrhea was found. Serum Calcium, FSH, LH, estradiol, progesterone, and prolactin were within normal range. Breast ultrasound revealed enlarged glands with zones of necrosis and ductal dilatation. Diagnosis of juvenile gigantomastia was made. She underwent free nipple graft reduction mammoplasty, with resection of 4450 g and 3850 g from the right and left breasts respectively. Pathology confirmed the macromastia. She relapsed 4 months after having her menarche, ie 8 months later, for which she had another reduction mammoplasty. Juvenile gigantomastia is a rare condition, in which local hypersensitivity for estrogen has been suggested. Surgical treatment is either reduction mammoplasty or subcutaneous mastectomy.

**Figure 1:**
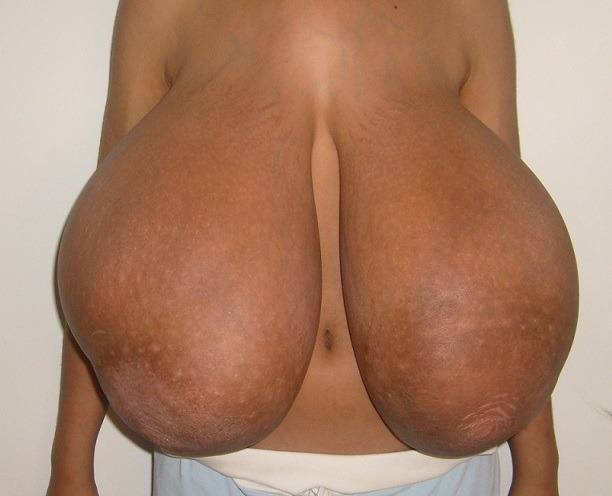
A) Front view of the gigantomastia; B) Lateral view

